# THE RELATIONSHIP BETWEEN ATTACHMENT, RELATIONSHIP QUALITY, AND DAILY STRESS AMONG DEMENTIA CAREGIVERS

**DOI:** 10.1093/geroni/igac059.3107

**Published:** 2022-12-20

**Authors:** Paulina Wuestefeld, Paige Goodwin, Robert Intrieri

**Affiliations:** Western Illinois University, Macomb, Illinois, United States; Western Illinois University, Macomb, Illinois, United States; Western Illinois University, Macomb, Illinois, United States

## Abstract

The current study assesses the relationship between attachment style, relationship quality, and daily stress among family caregivers of persons with dementia. The study incorporates a longitudinal burst design. In an initial meeting with the investigator, participants completed measures of attachment style (Experiences in Close Relationships Questionnaire Revised), past and current relationship quality (RQ), emotion regulation (Emotion Regulation Questionnaire), and caregiver burden (Zarit Burden Interview). For the next 14 days, participants were sent a link to a brief survey that included the Positive Affect and Negative Affect Schedule and the Perceived Stress Scale. They were also asked to estimate what proportion of their daily stress did they attribute to caregiving. There are significant positive correlations between caregiver burden and current negative RQ, r = 0.70, p = .008, as well as between attachment anxiety and current negative RQ, r = 0.60, p = .029. According to regression analyses, attachment anxiety predicts negative current RQ, R2 = .37, F(1, 11) = 6.32, p = .029. Current negative RQ predicts caregiver burden, R2 = .49, F(1, 11) = 10.55, p = .008. Additional analyses will explore the link between attachment style, emotion regulation, burden, relationship quality, and longitudinal assessments of daily stress and affect. Though preliminary, study results suggest that beyond caregiver burden, daily stress and well-being of caregivers is impacted by the relationship between attachment, relationship quality, and emotion regulation.

